# Open-Shell Variant of the London Dispersion-Corrected Hartree–Fock Method (HFLD) for the Quantification and Analysis of Noncovalent Interaction Energies

**DOI:** 10.1021/acs.jctc.1c01295

**Published:** 2022-02-15

**Authors:** Ahmet Altun, Frank Neese, Giovanni Bistoni

**Affiliations:** † Max-Planck-Institut für Kohlenforschung, Kaiser-Wilhelm-Platz 1, D-45470 Mülheim an der Ruhr, Germany; ‡ Department of Chemistry, Biology and Biotechnology, University of Perugia, 06123 Perugia, Italy

## Abstract

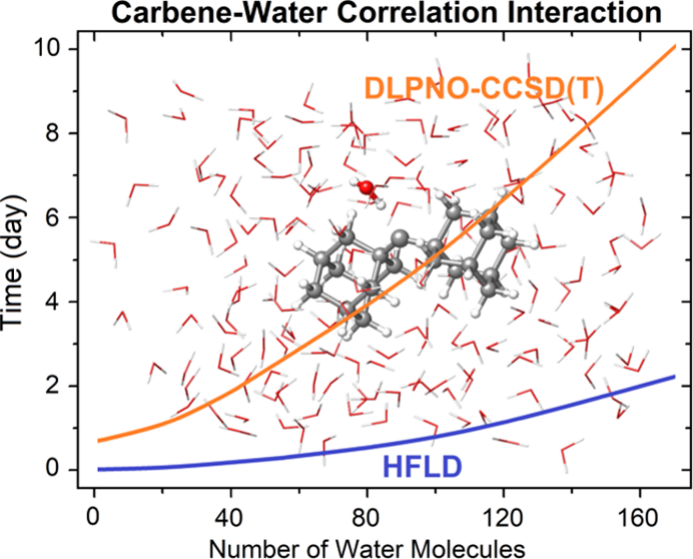

The London dispersion (LD)-corrected Hartree–Fock (HF) method (HFLD) is an *ab initio* approach for the quantification and analysis of noncovalent interactions (NCIs) in large systems that is based on the domain-based local pair natural orbital coupled-cluster (DLPNO-CC) theory. In the original HFLD paper, we discussed the implementation, accuracy, and efficiency of its closed-shell variant. Herein, an extension of this method to open-shell molecular systems is presented. Its accuracy is tested on challenging benchmark sets for NCIs, using CCSD(T) energies at the estimated complete basis set limit as reference. The HFLD scheme was found to be as accurate as the best-performing dispersion-corrected exchange-correlation functionals, while being nonempirical and equally efficient. In addition, it can be combined with the well-established local energy decomposition (LED) for the analysis of NCIs, thus yielding additional physical insights.

## 1 Introduction

Mean-field electronic structure theories like Hartree–Fock (HF) and density functional theory (DFT) are incapable of properly describing long-range correlation effects like the London dispersion (LD) energy.^1^ This often leads to large errors in the calculation of interaction energies, especially for systems held together by noncovalent interactions (NCIs),^2−16^ and stimulated the development of dispersion-corrected mean-field theories, which have found widespread applications in (bio)chemical research.^17−21^

For example, several Minnesota functionals have been internally parameterized to approximately account for LD effects.^22−26^ Alternatively, force field-like dispersion correction terms are added on top of the HF and DFT energies, as it is done in the popular HF-D or DFT-D method of Grimme and co-workers.^27−30^ The efficient small basis set composite “3c” variants of such approaches, namely, HF-3c^17^ and DFT-3c (*e.g*., B97–3c, PBEh-3c, HSE-3c, and r^2^SCAN-3c)^31−34^ methods, include additional geometrical counterpoise and short-range basis set incompleteness corrections. A conceptually similar approach to DFT-D is the Tkatchenko–Scheffler (TS) scheme,^35^ which relies on reference data for the free atoms for the calculation of the dispersion correction.^35−37^ Finally, vdW-density functional (vdW-DF)^38,39^ methods include a density-dependent term, *e.g.*, the VV10 nonlocal (NL) correlation functional that accounts for the dispersion energy.^40,41^ VV10 is included in “combinatorially” optimized exchange-correlation functionals, such as B97M-V,^42^ ωB97M-V,^43^ and ωB97X-V,^44^ and it is also used in the so-called HF-NL and DFT-NL methods.^45,46^

*Ab initio* variants of these approaches were also formulated. For example, HF interaction energies were corrected with dispersion terms obtained from the symmetry-adapted perturbation theory (SAPT),^47−53^ leading to the so-called dispersion-corrected HF methods (HFD).^54−56^ Analogously, HF and DFT interaction energies were also corrected with effective fragment potential (EFP)-derived dispersion terms, leading to HF-D(EFP) and DFT-D(EFP) methods.^57^

Unlike mean-field theories, correlated wave function-based methods naturally describe LD and can thus be used for computing NCI energies accurately within a supermolecular approach. In particular, the coupled-cluster method with singles, doubles, and perturbative treatment of triple excitations, *i.e*., CCSD(T), is known to provide extremely accurate results for a broad range of different chemical systems.^58^ By exploiting the rapid decay of electron correlation with the interelectronic distance, low scaling local variants of this approach have been developed.^59−61^ Among them, the domain-based local pair natural orbital CCSD(T) method, *i.e*., DLPNO-CCSD(T), has proven instrumental in a large number of chemical applications.^61−70^ It combines great efficiency^70−73^ with essentially canonical CCSD(T) accuracy. For relative energies, it reproduces the results of its canonical parent method within 1 kJ/mol when used in conjunction with the recently devised “complete PNO space” (CPS) extrapolation scheme, CPS(6/7),^74^ as shown on the most challenging sets of GMTKN55^30^ benchmark superset. In addition, the DLPNO-CCSD(T) approach can be combined with the well-established local energy decomposition (LED) scheme for the study of NCIs.^75−77^ This scheme decomposes the DLPNO-CCSD(T) interaction energy into physically meaningful fragment-pairwise energy terms, such as electrostatics, exchange, electronic preparation, LD, and nondispersive correlation. These terms were found to correlate reasonably well with those obtained using SAPT, especially in the weak-interaction limit.^78^ For these reasons, the DLPNO-CCSD(T)/LED methodology has found widespread applications in the study of intermolecular interactions.^75−86^

Importantly, the LED methodology can be used to identify the “dispersion excitations” in the DLPNO-CCSD correlation energy.^75−77,79^ By exploiting the multilevel implementation of the DLPNO-CCSD(T) method,^87^ one can solve the coupled-cluster equations while neglecting the nondispersive excitations, which leads to a cost-effective LD-corrected HF method called HFLD.^88^ On various closed-shell benchmark sets for NCIs, this approach typically provides sub-kcal/mol accuracy, as demonstrated on noble-gas dimers, as well as on the S66,^89^ L7,^90^ and LP14^83^ benchmark sets. Interaction energies computed with HFLD typically lie between those computed at the CCSD and CCSD(T) levels of theory.^88^ Therefore, this cost-effective approach can be used to study intermolecular interactions accurately in large and complex (bio-)molecular systems. For example, a recent combined HFLD/LED study^91^ on a large DNA duplex model (1001 atoms and 13 998 contracted basis functions) provided an in-depth characterization of the key inter- and intra-strand interactions responsible for the stability of human DNA.

**Figure 1:**
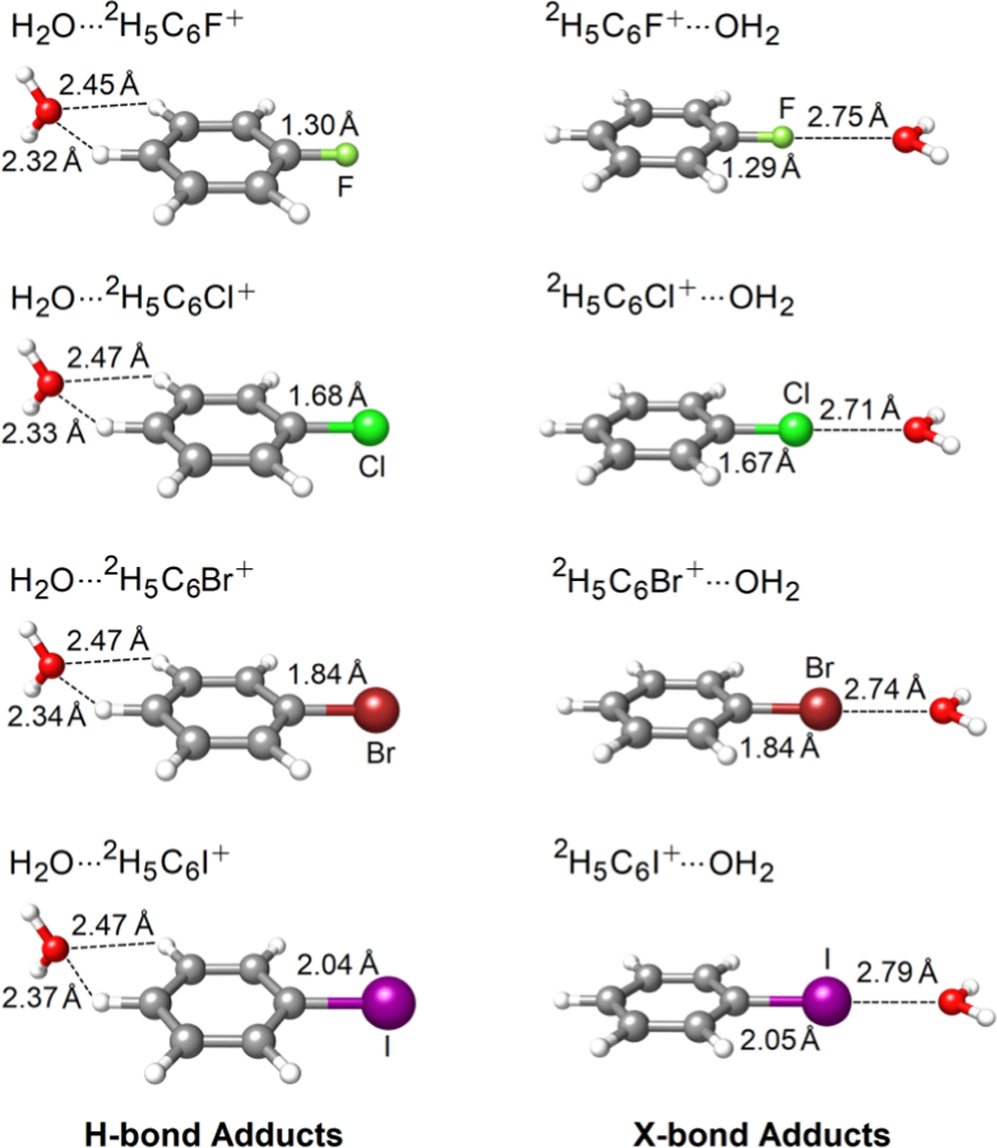
IB8 benchmark set of B3LYP-D3(BJ)/def2-TZVP-level optimized ionic H-bond and ionic X-bond adducts of ^2^H_5_C_6_X^+^ (X = F, Cl, Br, and I) with water.

**Figure 2:**
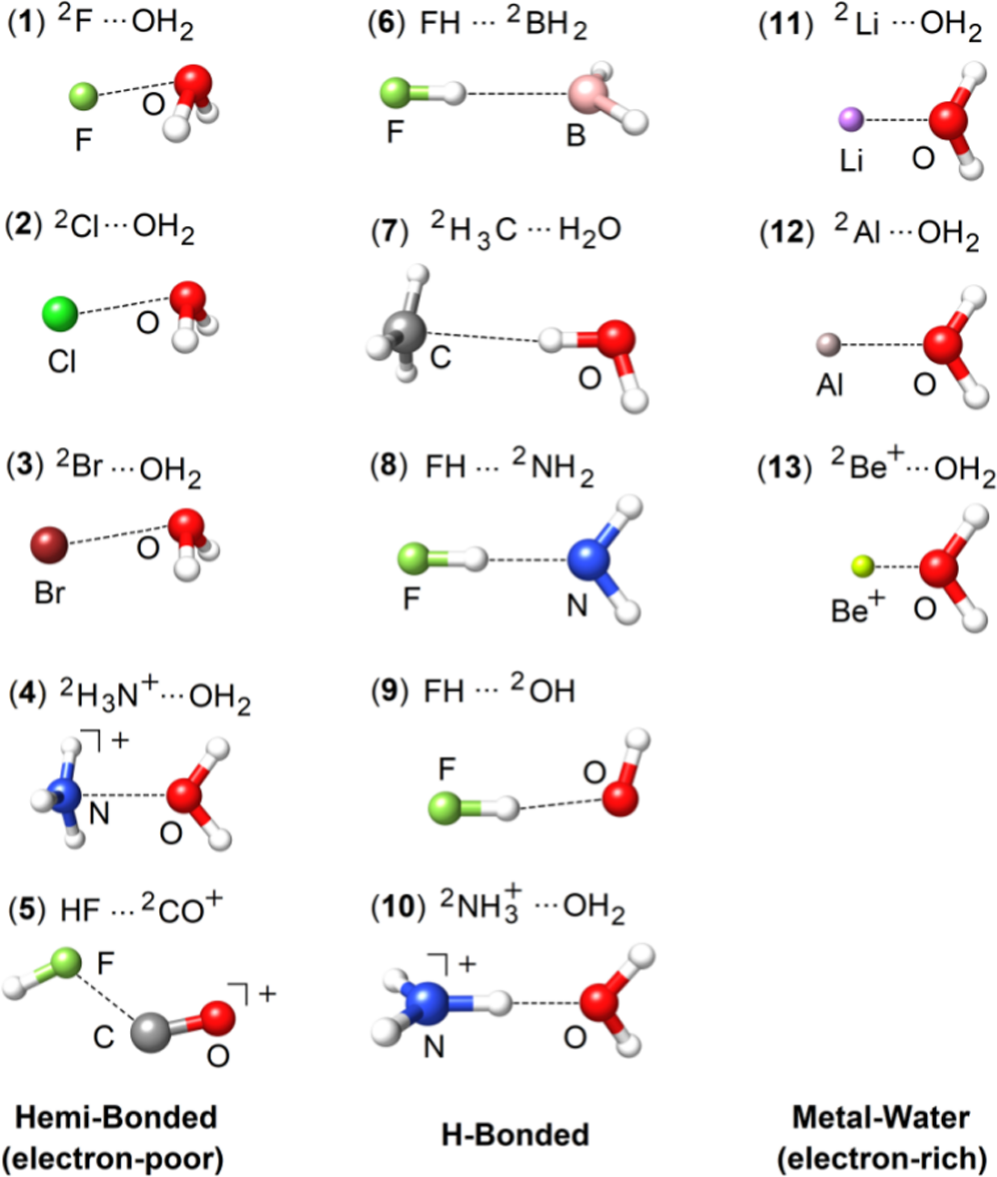
TA13 benchmark set of radical–solvent binary complexes and labeling of their molecular adducts, *i.e.*, **1**–**13**.

**Figure 3:**
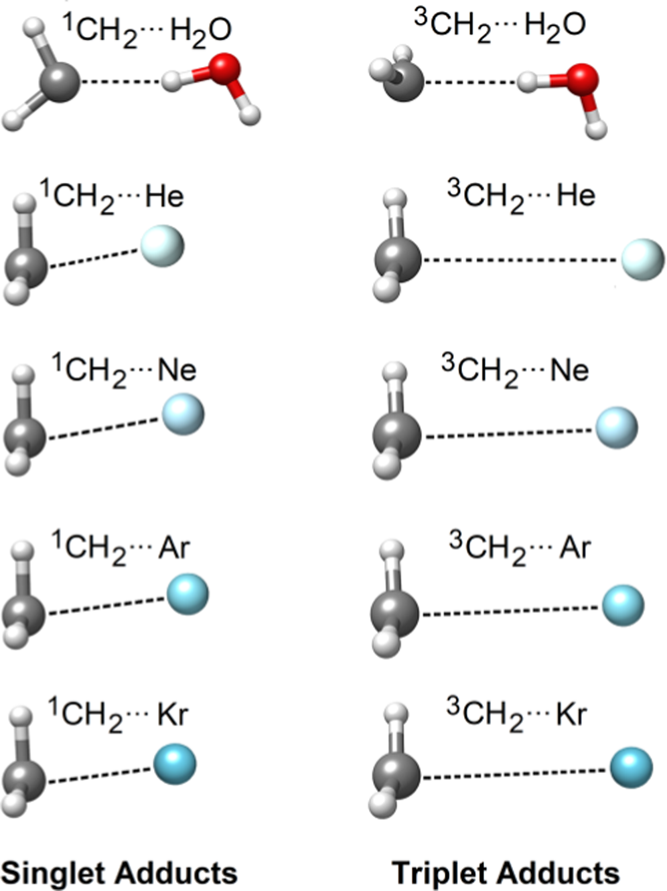
CARB10 benchmark set of neutral CH_2_ (singlet and triplet states) with noble gases (He, Ne, Ar, and Kr) and water.

**Figure 4:**
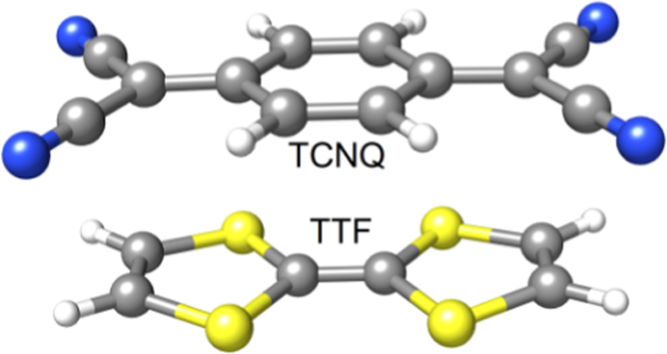
TTF-TCNQ ion pair.

In this study, we present an extension of the HFLD method to open-shell molecular systems. Its accuracy is assessed using accurate CCSD(T) results as a reference on several benchmark sets for NCIs, namely:

(i) A set of ionic bonded structures involving interactions between halogen-substituted benzene radical cations (^2^H_5_C_6_X^+^, X = F, Cl, Br, and I) and water^92,93^ (see Figure 1). This set is labeled in this study as IB8. A combined mass-selected ion spectroscopy and DFT study^92^ demonstrated that the binding motif (ionic H-bond *vs* ionic X-bond) of ^2^H_5_C_6_X^+^ is sensitive to the halogen atom X (F, Cl, Br, and I).
(ii) The TA13 set^94^ of radical–solvent binary complexes in the MGCDB84 database.^26^ This set encompasses electron-poor hemi-bonded (**1**–**5** in Figure 2) and H-bonded (**6**–**10** in Figure 2) interactions of several small neutral/cationic radicals with water (H_2_O) and hydrogen fluoride (HF). It also includes molecular complexes between electron-rich main group metal radicals and water (**11**–**13** in Figure 2). The accurate description of the NCIs in this set is extremely difficult with DFT, mostly due to the self-interaction error (SIE).^26,93^
(iii) The CARB10 set, consisting of molecular adducts between the simplest carbene, *i.e.*, methylene (CH_2_), and water/noble gases (He, Ne, Ar, and Kr) (see Figure 3). The interaction energies in this set were already studied using the DLPNO-CCSD(T)/LED scheme in a previous publication from our group.^76^
(iv) The interaction energy of the tetrathiafulvalene···tetracyanoquinodimethane (TTF-TCQN) ion pair (Figure 4) in its neutral (**0**) as well as one-electron (**1**) and two-electron (**2**) oxidized forms. Due to their high electrical conductivities, such structures have attracted great attention in molecular electronics.^95^

**Figure 5:**
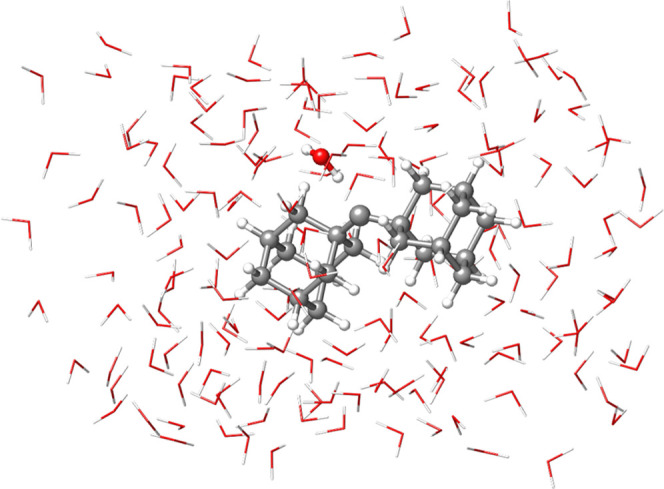
Di(1-adamantyl)carbene (DAC) in water.

The paper is organized as follows. The theory of the open-shell HFLD scheme is outlined in Section 2, while the computational details are provided in Section 3. The accuracy of the HFLD scheme is discussed in comparison with that obtained for various second-order Møller–Plesset perturbation theory MP2 variants as well as with several dispersion-corrected HF and DFT methods in Sections 4.1–4.4. A summary of the overall performance of the tested schemes is given in Section 4.5. Finally, the efficiency of the HFLD scheme and complementary analysis tools are discussed in Section 4.6 on a system of 561 atoms, in which the di(1-adamantyl)carbene^96^ (DAC) is solvated at its triplet state by 170 water molecules (see Figure 5). Section 5 is devoted to the concluding remarks of the study.

## 2 Theory

### 2.1 Open-Shell HFLD

In the following, only the aspects relevant to the open-shell algorithm of the HFLD method are discussed. The detailed description of its closed-shell variant can be found in ref (88).

In the UHF-DLPNO-CCSD framework, a restricted reference determinant is used, *i.e.*, the quasi-restricted orbitals (QROs)^97^ determinant or the restricted open-shell HF (ROHF) determinant. After solving the unrestricted open-shell coupled-cluster equations, the correlation energy is written as a sum of electron-pair correlation energy contributions.^61−70^ Using local second-order many-body perturbation theory, they can be divided into “weak pairs” (WP), with expected negligible contribution, and “strong pairs” (SP). While the SP are treated at the coupled-cluster level, the WP contribution is kept at the second-order level. The dominant SP contribution in the open-shell formalism reads
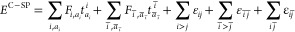
1where the indices *i* and *j* denote localized α spin orbitals, and *i* and *j̅* denote localized β spin orbitals. The first two terms represent the contribution from the single excitations, with *a*_*i*_ and *t*_*a*_*i*__^*i*^ being the singles PNOs and their amplitudes, respectively. The ε_*ij*_, ε_*i* *j̅*_, and ε_*i* *j̅*_ terms, *i.e.*, αα, ββ, and αβ pair correlation energies, respectively, can be written as a sum of double-excitation contributions
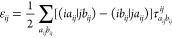
2
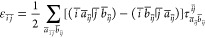
3
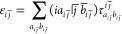
4where *a*_*ij*_ and *b*_*ij*_ are the PNOs for the *ij* pair; (*ia*_*ij*_|*jb*_*ij*_) are the two-electron integrals in Mulliken notation; and τ_*a*_*ij*_*b*_*ij*__^*ij*^ = *t*_*a*_*ij*_*b*_*ij*__^*ij*^ + *t*_*a*_*ij*__^*i*^*t*_*b*_*ij*__^*j*^ – *t*_*b*_*ij*__^*i*^*t*_*a*_*ij*__^*j*^ and  are the cluster amplitudes in the PNO basis, in which *t*_*a*_*ij*_*b*_*ij*__^*ij*^ and *t*_*a*_*ij*__^*i*^/*t*_*b*_*ij*__^*i*^ are the doubles and singles amplitudes of the coupled-cluster equations, respectively.

**Figure 6:**
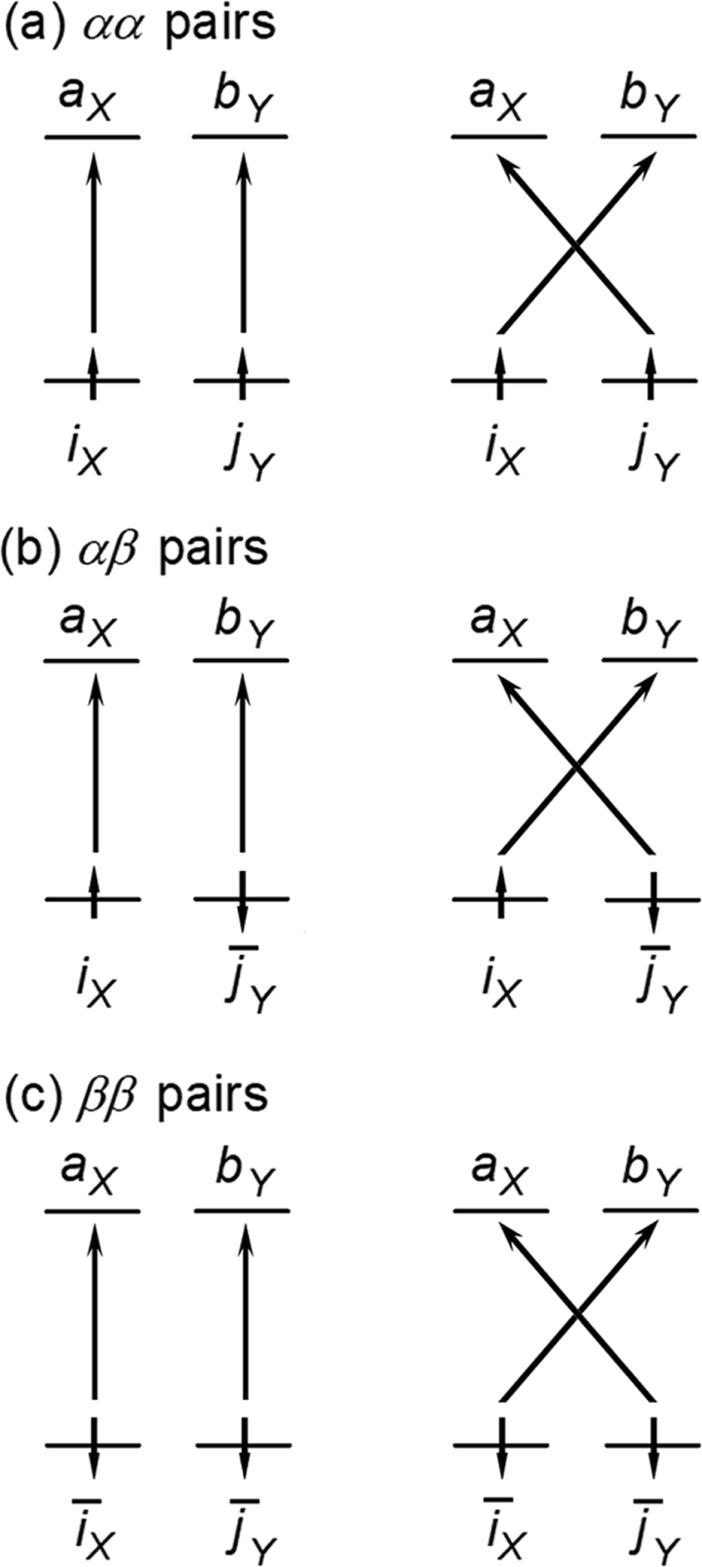
For a molecular adduct *XY*, genuine (left) and exchange (right) dispersion excitations of strong electron pairs from occupied orbitals to virtual orbitals (PNOs) in the framework of open-shell DLPNO-CCSD/LED scheme, where *i* and *j* denote localized α spin orbitals; *i̅* and *j̅* denote localized β spin orbitals; and *a* and *b* denote the corresponding PNOs. The subscripts *X* and *Y* denote the fragments in which the orbitals are localized.

The localized occupied orbitals can be assigned to the fragment in which they are dominantly localized. Thus, pair correlation energies can be divided into intrafragment pairs, for which both occupied orbitals are assigned to the same fragment, and interfragment pairs, for which the occupied orbitals are assigned to different fragments. In the LED framework, the PNOs are also assigned onto fragments. This allows us to divide the double excitations in the correlation energy into different families with different physical interpretation, such as intrafragment excitations, dynamic charge transfer excitations from intra- and interfragment pairs, and dispersion excitations.^75−77^ The all-important dispersion excitations for an adduct *XY* are schematically shown in Figure 6.

For example, for the αα pairs, the corresponding dispersion energy *E*_disp_^C-SP^(αα) reads^76^
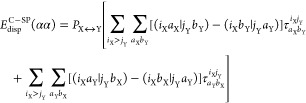
5where the subscripts *X* and *Y* are used to identify the fragments in which the orbitals are localized.

In the first step of the HFLD method,^88^ only the interfragment pairs are considered while solving the unrestricted open-shell CCSD equations. All of the remaining correlated pairs are neglected. Then, the LED analysis is used to single out the dispersion contribution from the other interfragment correlation terms. The interfragment part of WP contributions is essentially dispersive in nature and can thus be added to the SP dispersion to estimate the overall London dispersion (LD) energy at the CCSD level, *i.e.*, *E*_disp_^C-CCSD^. The HFLD interaction energy Δ*E*_int_^HFLD^ is then the sum of reference interaction energy Δ*E*_int_^ref^ and *E*_disp_^C-CCSD^
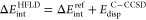
6

### 2.2 Choice of Reference Wave Function

As mentioned above, HFLD relies on a restricted reference determinant for the calculation of the LD energy *E*_disp_^C-CCSD^. If the QRO determinant is used as a reference, the reference interaction energy Δ*E*_int_^ref^ in eq 6 can, in principle, be computed at the UHF or QRO levels. This leads to two different schemes, namely, the QRO/HFLD and UHF/HFLD. Alternatively, the restricted open-shell HF (ROHF) determinant can be used as a reference in HFLD calculations, which leads to the ROHF/HFLD approach. Importantly, the LD energy is only weakly affected by the choice of the reference wave function, as shown in the “benchmark set name-HFLD” sheets in the Supporting Information.

The mean absolute error (MAE) obtained for HFLD/TightPNO/CBS(3/4) (with *T*_CutPNO_ = 10^–8^) for all benchmark sets with different reference schemes is provided in Table 1.

**Table 1:** Mean Absolute Error (kcal/mol) of HFLD/TightPNO/CBS(3/4) Interaction Energies with *T*_CutPNO_ = 10^–8^ for the Individual Benchmark Sets as well as for All Molecules in These Sets Using UHF, QRO, and ROHF in the Reference Part Relative to the Present (DLPNO-)CCSD(T)/CBS Benchmark Values^a^

scheme	IB8	TA13	CARB10	TTF-TCNQ	overall
UHF/HFLD	0.20	1.78	0.39	1.14	0.94
QRO/HFLD	0.90	1.74	0.40	1.16	1.10
ROHF/HFLD	0.54	1.79	0.39	1.25	1.04

a See Section 3 for the CCSD(T)/CBS benchmark settings of IB8, TA13, and CARB10 sets and for the DLPNO-CCSD(T)/CBS benchmark settings of the TTF-TCNQ set.

For the ionic interactions in the IB8 set, the error in the HFLD interaction energies is significantly smaller for UHF/HFLD than with QRO/HFLD and ROHF/HFLD. For the other sets, HFLD provides essentially the same accuracy irrespective of the reference used. Therefore, only UHF/HFLD results are discussed in the following, and this scheme is also selected as the default in open-shell HFLD calculations in ORCA. Notice that, for cases with significant spin contamination, UHF/HFLD might provide larger errors than ROHF/HFLD or QRO/HFLD. This is the case, for instance, for the ^2^CO^+^ monomer of reaction 5 in the TA13 set (see Figure 2). For this molecule, the computed ⟨**S**^2^⟩ shows a deviation larger than 0.2 from the value expected for a doublet. This leads to larger errors in the computed interaction energies for reaction 5, namely, 4.13, 2.19, and 2.90 kcal/mol for UHF/HFLD, QRO/HFLD, and ROHF/HFLD references, respectively. For the sake of comparison, it is worth mentioning that similar results concerning the impact of the reference determinant used were found at the semiempirical HF-D level, as shown in the “benchmark set name-DFT-HFD” sheets in the Supporting Information.

### 2.3 Accuracy Considerations

The HFLD scheme is an efficient approach for the study of NCIs in large molecular systems, and it does not require any empirical parameter. Its great efficiency is achieved by neglecting the intrafragment pairs while solving the unrestricted open-shell coupled-cluster equations. The triples correction (T) is also neglected. These approximations might, in principle, lead to different errors in interaction energy calculations:

(i) By neglecting the coupling between intra- and interfragment correlation pairs, one introduces an approximation in the calculation of *E*_disp_^C-CCSD^. However, the effect of this approximation has been shown to be relatively small.^88^
(ii) The nondispersive correlation contribution Δ*E*_no-disp_^C-CCSD^ to the interaction energy provides a correction to all contributions to the interaction that are only approximately described at the HF level, such as the permanent and induced electrostatics.^77^ The accuracy of HFLD rests, to a large extent, on the cancellation between Δ*E*_no-disp_^C-CCSD^ and the triples correction contribution to the interaction energy, Δ*E*_int_^C-(T)^. As discussed in the original HFLD paper,^88^ these terms might not cancel out in systems with large Δ*E*_no-disp_^C-CCSD^ contributions, *i.e.*, systems whose electronic structure is poorly described at the HF level. All dispersion-corrected HF methods are expected to fail in these situations.

**Figure 7:**
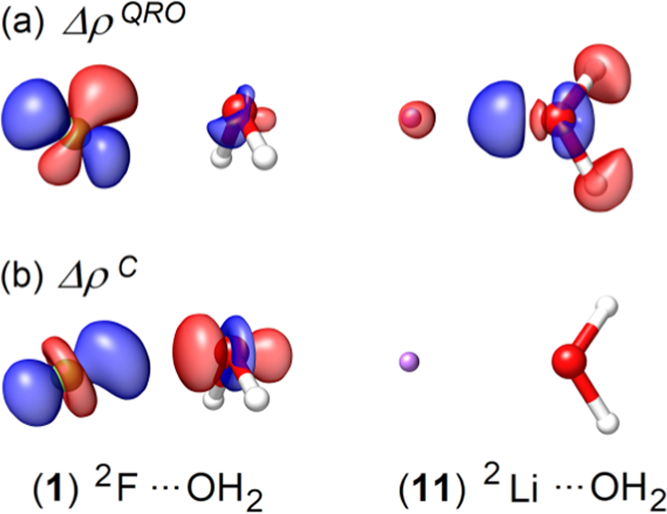
Contour plots of the (a) reference Δρ^QRO^ and (b) correlation Δρ^C^ parts of the one-electron density deformation function (Δρ) associated with the ^2^F···OH_2_ (**1**, left) and ^2^Li···OH_2_ (**11**, right) bonds in the TA13 set. Δρ^QRO^ was computed at the QRO/aug-cc-pV5Z level. Δρ^C^ represents the contribution from the DLPNO-CCSD/TightPNO/aug-cc-pV5Z correlation to Δρ. All plots are given with density isosurface contour values of ±0.003 *e*/bohr^3^. Blue and red surfaces identify regions of electron density accumulation and depletion, respectively.

The latter point deserves to be discussed in more detail using two illustrative examples: the ^2^F···H_2_O (**1** in the TA13 set) and the ^2^Li···H_2_O (**11** in the TA13 set) interactions. For these interactions, Figure 7 shows the contour plots of the reference (Δρ^QRO^) and correlation (Δρ^C^) parts of the corresponding one-electron density deformation function (Δρ), computed at the DLPNO-CCSD level. Δρ represents the charge rearrangement taking place upon the formation of the hemi-bond in ^2^F···H_2_O and of the metal–water bond in ^2^Li···H_2_O (note that Δρ = Δρ^QRO^ + Δρ^C^; see ref (76) for details).

**Figure 8:**
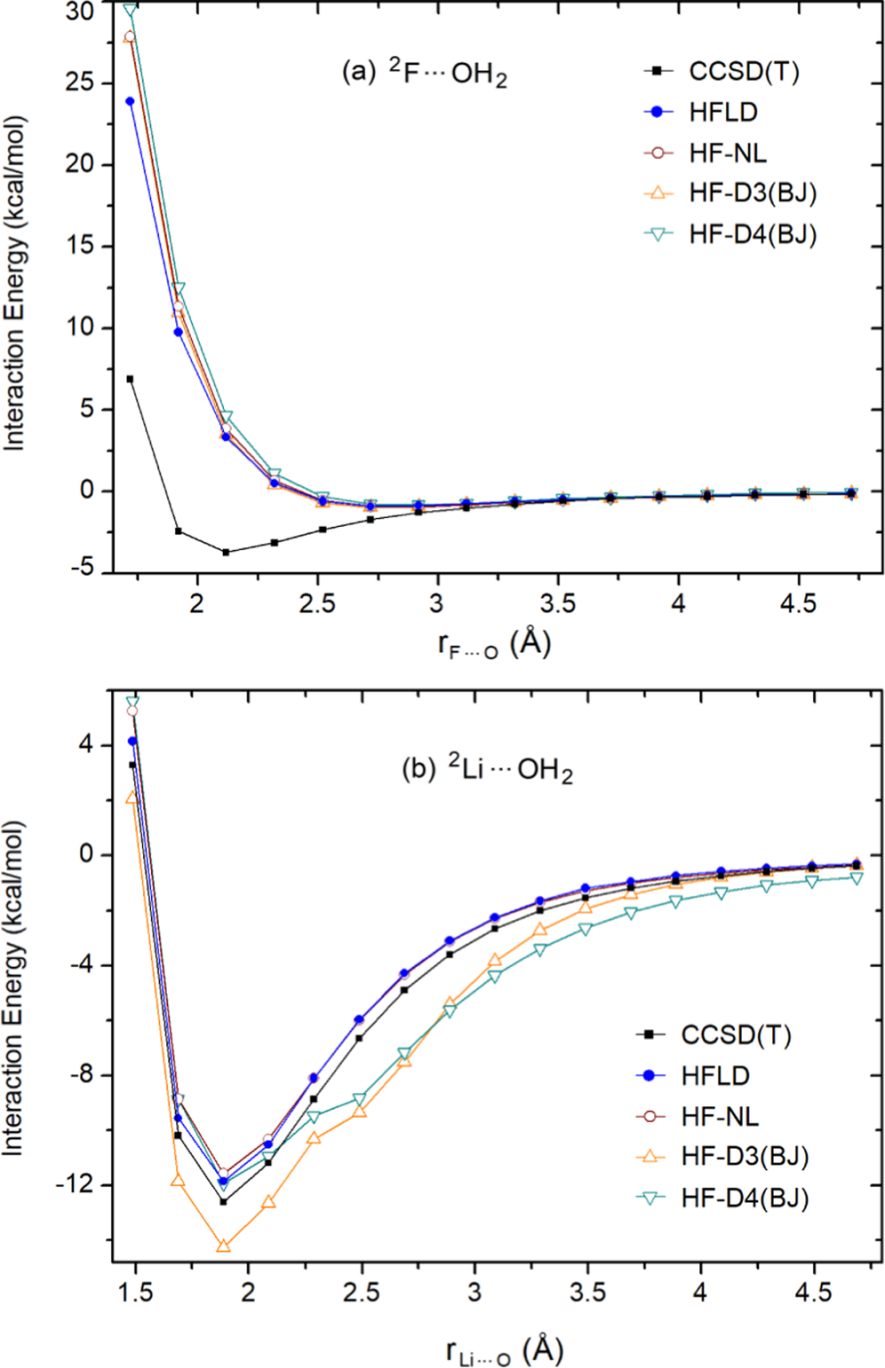
Interaction energy profile of (a) ^2^F···OH_2_ (**1** in the TA13 set) as a function of fluorine–oxygen distance and of (b) ^2^Li···OH_2_ (**11** in the TA13 set) as a function of the metal-oxygen distance, computed at various levels of dispersion-corrected Hartree–Fock theory [HFLD/TightPNO/CBS(3/4) (with *T*_CutPNO_ = 10^–8^), HF-NL/aug-cc-pV5Z, HF-D3(BJ)/aug-cc-pV5Z, and HF-D4(BJ)/aug-cc-pV5Z]. Accurate CCSD(T)/CBS(3/4) energy profiles are also shown for comparison. In all cases, the geometry of the water molecule was frozen to that it has at the equilibrium geometry of **1** (a) and **11** (b).

At the reference QRO level, for the hemi-bonded adduct **1**, the interaction polarizes mainly the π orbitals of the F atom, without any noticeable charge transfer between F and water. However, at the DLPNO-CCSD level, a significant amount of π electron density is transferred from water to the F atom. This picture is consistent with the Mayer bond order of the F···O hemi-bond at QRO (0.00 *e*) and DPNO-CCSD (0.15 *e*) levels. Hence, electron correlation significantly changes the nature of the F···O interaction in **1**, increasing its “charge transfer” nature. This is reflected in the large value assumed by Δ*E*_no-disp_^C-CCSD^ in this system in its equilibrium structure (−4.5 kcal/mol). Since this correlation term is not considered in dispersion-corrected HF methods, they all fail badly for this system, as clearly shown in Figure 8a.

In contrast, for the metal–water adduct **11**, electron correlation has virtually no effect on the electron density deformation. Thus, it is not surprising that the nondispersive correlation contribution to the interaction is negligibly small (Δ*E*_no-disp_^C-CCSD^ = +0.05 kcal/mol at the equilibrium). In this case, the correlation interaction energy is dominated by the dispersion term (*E*_disp_^C-CCSD^ = −1.72 kcal/mol at the equilibrium). Hence, dispersion-corrected HF methods are expected to perform well for such adducts. As shown in Figure 8b, this is actually the case for HFLD and HF-NL for the entire section of the potential energy surface investigated. In contrast, HF-D3(BJ) significantly overestimates interaction energies at all distances, while HF-D4(BJ) performs very well around the equilibrium geometry but fails in the medium–long range.

This example clearly shows that the magnitude of the correlation interaction energy is influenced by different physical effects besides LD, as it was previously discussed by us in a recent work.^77^ This constitutes a significant limitation to all dispersion-corrected HF methods.

## 3 Computational Details

All calculations were carried out with a development version of the ORCA program package based on version 5.0.^98,99^ The default frozen core settings in ORCA were used in all coupled-cluster, HFLD, and MP2 calculations.^100^ The presently introduced open-shell HFLD method is available free of charge starting from the 5.0 release of ORCA. ORCA input file specifications for all used methods and benchmark sets were collected in the “INPUT” sheet in the Supporting Information.

### 3.1 Geometries

The Cartesian coordinates of all structures investigated in this study, including the previously published ones, were collected in the “XYZ” sheet in the Supporting Information.

For the adducts in the IB8 set, geometries were computed at the B3LYP^101−103^ level of theory by incorporating the D3 dispersion correction with Becke–Johnson (BJ) damping.^27^ The def2-TZVP basis set was used for all atoms.^104,105^

The geometries used for the adducts in the TA13 and CARB10 sets were previously obtained from geometry optimizations at the CCSD(T)/aug-cc-pVTZ^94^ and DLPNO-CCSD(T)/TightPNO/aug-cc-pVTZ^76^ levels, respectively. The structure of the TTF-TCNQ pair was taken from ref (106).

For DAC in water, an initial guess structure was built by placing DAC in a preequilibrated box of 170 water molecules. Then, geometry optimization was carried out at the quantum mechanical/molecular mechanical (QM/MM) level for the triplet state, using an electrostatic embedding scheme.^107^ The QM and MM portions include DAC (51 atoms) and water molecules (510 atoms), respectively. The QM and MM computations were performed at the B3LYP-D3(BJ)/def2-TZVP level within the RIJCOSX approximation^108,109^ and with the TIP3P model as implemented for the CHARMM force field,^110^ respectively.

### 3.2 Interaction Energy Calculations

At the above-described geometries, basis set superposition error (BSSE)-corrected^111^ interaction energies were computed with a number of dispersion-corrected HF and DFT methods as well as with HFLD, canonical CCSD(T), DLPNO-CCSD(T), and various MP2 variants. Unless stated otherwise, these calculations were performed with the standard aug-cc-pV*n*Z (*n* = D, T, Q, and 5) basis sets.^112−114^ For the calculations using the resolution of identity (RI) approximation,^115,116^ the corresponding auxiliary basis sets were generated with the automated auxiliary basis set construction module of ORCA (*i.e.*, “autoaux”), using the maximum possible angular momentum.^117^ For the Br and I atoms in the IB8 set, the core-valence cc-pwCV*n*Z-PP (*n* = D, T, Q, and 5) basis sets, combined with the SK-MCDHF-RSC effective core potential, were used.^118,119^ For these atoms, the standard matching auxiliary basis sets were used. In the following, these basis set settings will be simply called as *n*Z, *i.e.*, DZ, TZ, QZ, and 5Z. The results obtained with wave function-based correlation methods were also extrapolated to the complete basis set (CBS) limit, as described previously^62^ (see also CBS sheet in the Supporting Information for details).

#### 3.2.1 Semiempirical Dispersion-Corrected HF and DFT Calculations

Unless otherwise specified, all of the semiempirical dispersion-corrected HF and DFT calculations described in this section were performed in the UHF and unrestricted Kohn–Sham (UKS) frameworks, respectively.

In HF-D and DFT-D calculations,^45^ the D3^120^ and D4^29^ dispersion corrections were used in conjunction with either zero damping [D3(0)] or with Becke–Johnson damping [D3(BJ) and D4(BJ)].^27^ The charge-dependent D4 term was calculated with ATM charges. HF-D calculations were performed with BJ damping, resulting in HF-D3(BJ) and HF-D4(BJ) methods. The following DFT-D functionals were tested: BLYP-D3(0), BLYP-D3(BJ), BLYP-D4(BJ), B3LYP-D3(BJ), B3LYP-D4(BJ), M06–2X-D3(0), and ωB97M-D3(BJ).^26,30^ As M06-2X is parameterized internally for including dispersion effects, it performs already very well without the D3(0) correction. Hence, the results obtained using the M06-2X functional without any additional dispersion correction were also discussed.^25^

Among the methods that rely on the VV10 nonlocal correlation functional, HF-NL, BLYP-NL, B3LYP-NL, and ωB97M-V were selected.^26,30^ Importantly, ωB97M-V^43^ was found to be the overall best performer on the MGCDB84 benchmark set, which includes about 5000 molecules.^26^

For the sake of simplicity, only the accuracy of ωB97M-V, M06–2X, BLYP-D3(BJ), and B3LYP-D3(BJ) is discussed in the main manuscript for individual interaction energies. The comparison between B3LYP-D3(BJ) and BLYP-D3(BJ) was used to assess the effect of the SIE for these systems. For all other functional/dispersion correction combinations (see above), only the overall accuracy of the method is discussed in the main manuscript. The results obtained for all combinations, as well as the associated error statistics, can be found in the Supporting Information.

For all DFT and dispersion-corrected HF calculations, the 5Z basis set was used. For the IB8, TA13, and CARB10 benchmark sets, DFT calculations were all performed without RI or any other integral approximation. For the TTF-TCNQ pair, B3LYP calculations were carried out using the RIJK approximation,^121−124^ while the RIJCOSX approximation was used for all other functionals.^108,109^ Consistent with the HFLD settings (see below), the HF part of all HF-D3(BJ), HF-D4(BJ), and HF-NL calculations was computed using the RIJK approximation.

As representative of composite “3c” methods, the HF-3c^17^ (D3) and r^2^SCAN-3c^34^ (D4) schemes were also tested.

#### 3.2.2 MP2 Calculations

For each benchmark set, the accuracy of RI-MP2, spin component scaled RI-SCS-MP2, as well as their orbital optimized OO-RI-MP2 and OO-RI-SCS-MP2 variants, was assessed.^125−127^ Both UHF and ROHF references were tested for RI-MP2 and RI-SCS-MP2. In the reference part of the OO variants, only UHF was used due to the unavailability of OO-MP2 for ROHF references. Auxiliary basis sets for the correlation part were constructed as described at the beginning of Section 3.2 for each benchmark set. With TZ basis set settings, we demonstrated that the RI approximation does not introduce any error in the relative MP2 energies. Therefore, in the following, for the sake of simplicity, we drop the RI prefix in the name of these methods. In the manuscript, all MP2 calculations were carried out using CBS(3/4) extrapolation, consistent with the other correlation calculations performed in this study (see below). For the affordable cases, results with 5Z basis set were also reported in the “benchmark set name-MP2” sheets in the Supporting Information. No significant difference was found between the 5Z and the extrapolated CBS(3/4) results.

#### 3.2.3 Canonical CCSD(T) Calculations

For the TA13 and CARB10 sets, canonical CCSD(T) interaction energies at the estimated CBS(3/4) limit were used as benchmark. For the IB8 set, the TZ basis is the largest affordable for the (T) contribution. Therefore, CCSD/CBS(3/4) + (T)/CBS(2/3) composite CBS setting (denoted hereafter as CBS*) was considered for this set as the benchmark level. Notice that the (T) contributions computed with the TZ basis set and CBS(2/3) have a mean absolute deviation of just 0.02 kcal/mol (see the IB8-CAN sheet in the Supporting Information). Thus, CBS(2/3) is expected to provide nearly converged (T) contributions for this set.

These CCSD(T) calculations were performed without any RI approximation. Their reference energies were obtained at the quasi-restricted orbital (QRO) level.^97^

For the TA13 set, BSSE-uncorrected CCSD binding energies plus contributions up to (Q) excitations were already provided in the literature.^94^ However, for consistency with the settings used for the other benchmark sets for the reference calculations, we calculated BSSE-corrected CCSD(T) interaction energies also for this set. For completeness, geometrical preparation energies were also provided (see TA13-CAN and TA13-DLPNO sheets in the Supporting Information). The presently computed BSSE-corrected and the previous BSSE-uncorrected CCSD(T) energies are very similar to each other.

#### 3.2.4 DLPNO-CCSD(T) Calculations

QROs^74^ for the reference part were obtained within the RIJK approach.^121−124^ The augmented Hessian Foster-Boys (AHFB) scheme was employed for localizing occupied orbitals while the virtual orbitals (PNOs) were localized using the FB scheme in the LED calculations.^128^ Unless otherwise specified, the perturbative triples correction was calculated using the iterative (*T*_1_) algorithm. The semicanonical (*T*_0_) algorithm^129,130^ was used only for DAC in water.

Correlation energies computed with TightPNO settings (default *T*_CutPNO_ = 10^–7^)^61,70^ can be extrapolated to CPS limit as^74^
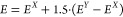
7where *E* is the estimated correlation energy at the CPS(*X*/*Y*) limit, while *E*^*X*^ and *E*^*Y*^ are the DLPNO-CCSD(T) correlation energies obtained with *T*_CutPNO_ = 10^–*X*^ and *T*_CutPNO_ = 10^–(*X*+1)^, respectively. CPS(6/7) extrapolation has been shown^74^ to provide sub-kJ/mol accuracy on the most challenging subsets of the GMTKN55 superset,^30^ and it retains essentially canonical CCSD(T) accuracy also for large systems.^131^ For the IB8, TA13, and CARB10 sets, the MAE associated with the DLPNO-CCSD(T)/CPS(6/7)/CBS(3/4) results (with respect to the canonical CCSD(T)/CBS benchmark data described above) is only 0.11, 0.08, and 0.04 kcal/mol, respectively. For the TTF-TCNQ pair, the present DLPNO-CCSD(T)/CPS(6/7)/CBS(3/4) interaction energies (see the TTF-TCNQ-DLPNO sheet in the Supporting Information) were used as the benchmark energies.^132,133^

It is worth mentioning here that the cost-effective CPS(5/6) extrapolation scheme has been shown to provide results with an accuracy that is similar to that obtained with *T*_CutPNO_ = 10^–7^ in most cases, while it typically fails for dispersion-bound systems.^74^ This is further confirmed in this study. While CPS(5/6) extrapolation fails for the TTF-TCNQ pair, it provides a reasonable accuracy for all other sets of this study (see the PNO-SIZE sheet in the Supporting Information for the summary of the results with different PNO settings).

#### 3.2.5 HFLD Calculations

As in the parent DLPNO-CCSD(T)/LED calculations, the AHFB and FB schemes^128^ were employed for localizing occupied orbitals and PNOs, respectively. To test the efficiency and accuracy of HFLD for NCIs of open-shell systems, two different computational settings were used and discussed in the manuscript.

(i) HFLD/TightPNO/CBS(3/4) with *T*_CutPNO_ = 10^–8^. This methodology provides results that are converged with respect to both the PNO settings and the basis set size in HFLD calculations, as summarized in the PNO-SIZE sheet in the Supporting Information. Hence, it is recommended for high-accuracy calculations. It is denoted hereafter as “the gold HFLD”. Unless otherwise specified, the RIJK approach was used in the reference part of these calculations.^121−124^ It should be mentioned here that systems with a strongly delocalized occupied orbital space, such as multicenter stacking interactions and metal–ligand systems with large electron sharing, typically span very large PNO spaces. For such systems, as the PNO space is very large, the localization of the PNO space with the FB scheme might be challenging when diffuse basis functions are used, especially with aug-cc-pV5Z and larger basis sets (a warning message is printed in ORCA if the localization of the PNO space fails in HFLD calculations). This makes the decomposition of the correlation energy into dispersive and nondispersive contributions challenging and might lead to significant errors. For these reasons, HFLD calculations with basis sets larger than aug-cc-pVQZ are not recommended for such systems. Also, it is important to mention that the various approximations used in HFLD make the dependence of the correlation energy with the size of the PNO space less smooth than in the parent DLPNO-CCSD method. Hence, CPS extrapolation approaches are not recommended with HFLD.
(ii) As a cost-effective alternative to the methodology described above, HFLD was tested in conjunction with NormalPNO* settings.^88^ These correspond to the standard NormalPNO settings used in DLPNO-CCSD(T) calculations but with the *T*_CutPairs_ threshold set to the more conservative value of 10^–5^ hartree.^61,70^ In the original HFLD paper,^88^ it was demonstrated that such NormalPNO* settings provide a very good balance between accuracy and computational cost. For these calculations, the def2-TZVP(-f) basis set was used for all atoms. Matching auxiliary basis sets were used in the SCF and correlation parts.^134^ As HFLD results show a very fast convergence with the basis set size (see the original HFLD paper,^88^ our DNA study,^91^ as well the PNO-SIZE sheet in the Supporting Information of this work), this basis set settings typically provide reasonably well-converged energetics. This cost-effective HFLD/NormalPNO*/def2-TZVP(-f) approach is denoted hereafter as HFLD*. Unless otherwise specified, the RIJCOSX approach,^108,109^ which is significantly more efficient than RIJK for large molecules,^78^ was used for the reference part in HFLD* calculations.

On the QM/MM structure of DAC in water, the efficiency of HFLD* interaction energy calculations was compared with that of DLPNO-CCSD(T_0_)/NormalPNO*/def2-TZVP(-f) by including different numbers of water molecules in the calculations. Both HFLD* and DLPNO-CCSD(T_0_) calculations were performed using (i) the RIJCOSX^108,109^ approach in the SCF part and (ii) the corresponding matching auxiliary basis sets of def2-TZVP(-f) in both reference and correlation parts.^134^

## 4 Results and Discussion

In this section, the accuracy of MP2, SCS-MP2, dispersion-corrected HF (HFLD, HF-3c, HF-D3(BJ), HF-D4(BJ), and HF-NL), and dispersion-corrected DFT methods (r^2^SCAN-3c, BLYP-D3(BJ), B3LYP-D3(BJ), M06-2X, and ωB97M-V) is compared on the above-mentioned benchmark sets. The efficiency of the HFLD scheme is also illustrated on a large system with 561 atoms (Figure 5). All of the calculated interaction energies and their errors statistics are provided in the Supporting Information. In the main text, we discuss the results in terms of MAEs. The mean errors (MEs) and root-mean-square errors (RMSEs) are provided in the Supporting Information.

### 4.1 Interaction Energies of the Adducts in the IB8 Set

**Figure 9:**
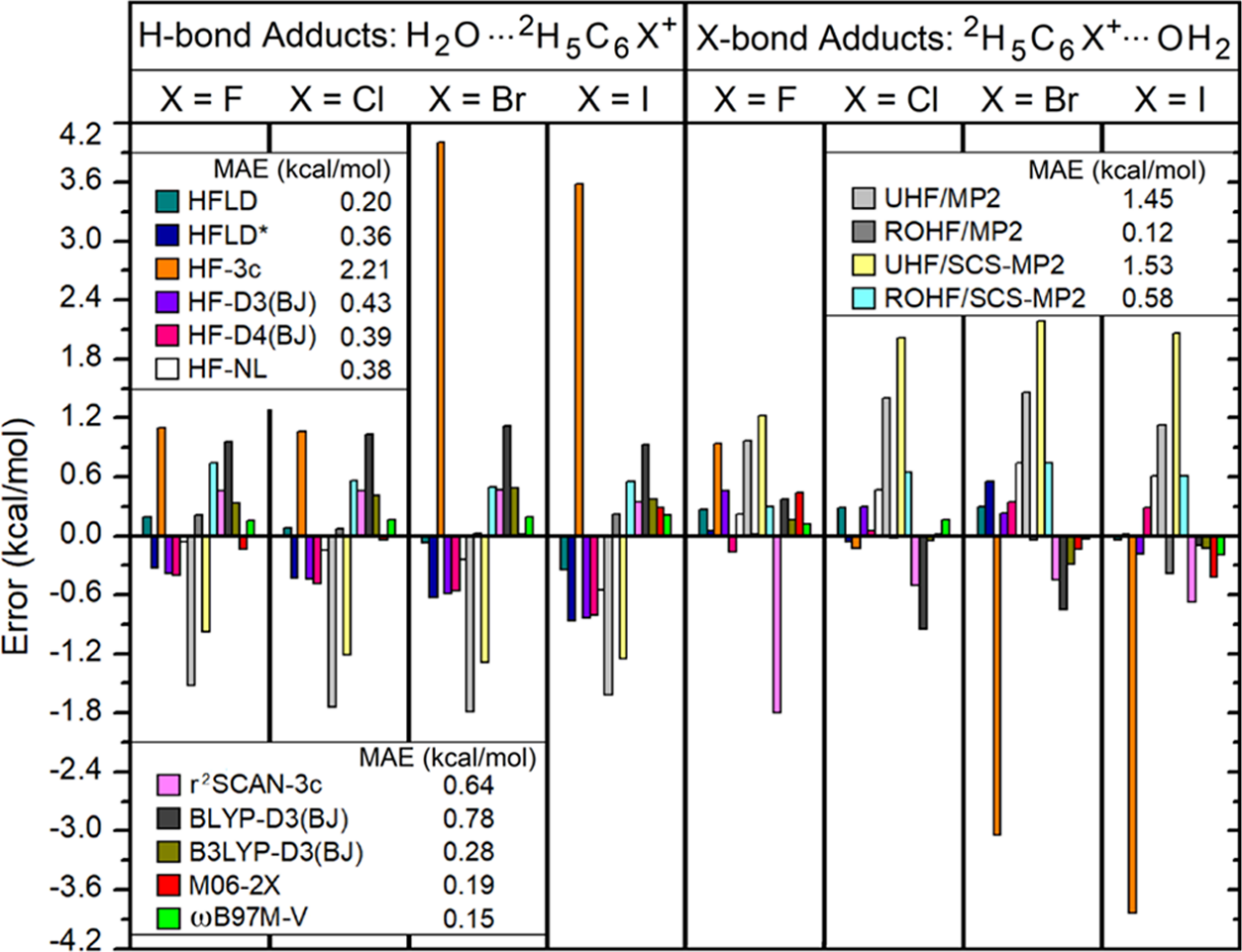
Error of interaction energy of each adduct in the IB8 set calculated with several dispersion-corrected HF and DFT methods as well as with MP2 and SCS-MP2 relative to the CCSD(T)/CBS* reference.

The interaction energy error of each adduct in the IB8 set calculated with MP2, SCS-MP2, and several dispersion-corrected HF and DFT schemes is as given in Figure 9 relative to the present CCSD(T)/CBS* reference. The IB8 set encompasses electrostatically dominated ionic H- and ionic X-bonds involving π systems (see Figure 1). For these adducts, LD energy contributions are noticeably large, varying typically between 1 and 3 kcal/mol (see the IB8-DLPNO and IB8-HFLD sheets in the Supporting Information).

For this set, gold HFLD settings provide an excellent accuracy (MAE = 0.20 kcal/mol), while the cost-effective HFLD* alternative shows slightly larger errors (MAE = 0.36 kcal/mol). ωB97M-V (MAE = 0.15 kcal/mol) and M06–2X (MAE = 0.19 kcal/mol) show an accuracy that is similar to that obtained with the gold HFLD settings, while B3LYP-D3(BJ) (MAE = 0.27 kcal/mol) and the other noncomposite dispersion-corrected HF methods provide slightly larger errors (MAE = ∼0.4 kcal/mol). BLYP-D3(BJ) and r^2^SCAN-3c functionals show significantly larger errors (MAE = ∼0.7 kcal/mol), while HF-3c seems to fail dramatically for this set (MAE = 2.21 kcal/mol). Finally, UHF/MP2 and UHF/SCS-MP2 also provide very large errors (MAE = ∼1.5 kcal/mol), while ROHF/MP2 shows an excellent performance for this set (MAE = 0.12 kcal/mol). Interestingly, the ROHF/SCS-MP2 error (MAE = 0.58 kcal/mol) is about 5 times worse than the ROHF/MP2 error for this set.

**Figure 10:**
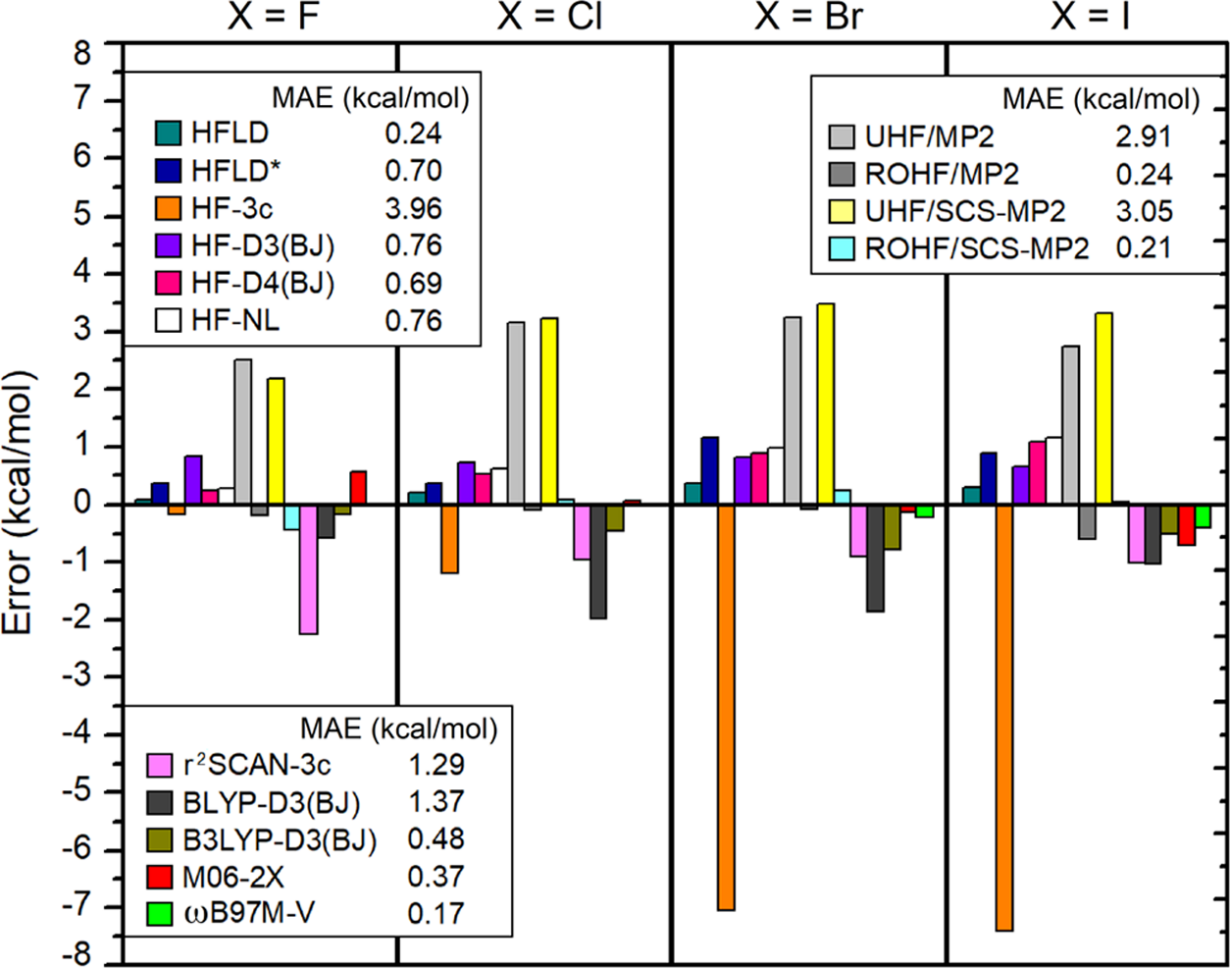
Error in the relative interaction energies between X-bonded and H-bonded adducts in the IB8 set calculated with several dispersion-corrected HF and DFT methods as well as with MP2 and SCS-MP2 in reference to the CCSD(T)/CBS* results.

Describing H-bonded and X-bonded adducts at nearly the same accuracy is important in trend studies. Therefore, we also assessed the accuracy of all methods in predicting relative interaction energies between X-bonded and H-bonded adducts (see Figure 10).

The errors in the relative interaction energies are typically larger than those just discussed. The overall best-performing functional ωB97M-V (MAE = 0.17 kcal/mol) provides an accuracy that is similar to that found with the gold HFLD settings (0.24 kcal/mol), while the error increases significantly with HFLD* and all other dispersion-corrected HF methods (∼0.70 kcal/mol). ROHF/MP2 (MAE = 0.24 kcal/mol), ROHF/SCS-MP2 (MAE = 0.21 kcal/mol), M06–2X (MAE = 0.38 kcal/mol), and B3LYP-D3(BJ) (MAE = 0.47 kcal/mol) provide analogous or comparable accuracies to HFLD. Therefore, all of these methods can be safely used in trend studies. By contrast, HF-3c (MAE = 3.96 kcal/mol), UHF/MP2, and UHF/SCS-MP2 (MAE = ∼3.0 kcal/mol) are not recommended in this case.

### 4.2 Interaction Energies of the Adducts in the TA13 Set

**Figure 11:**
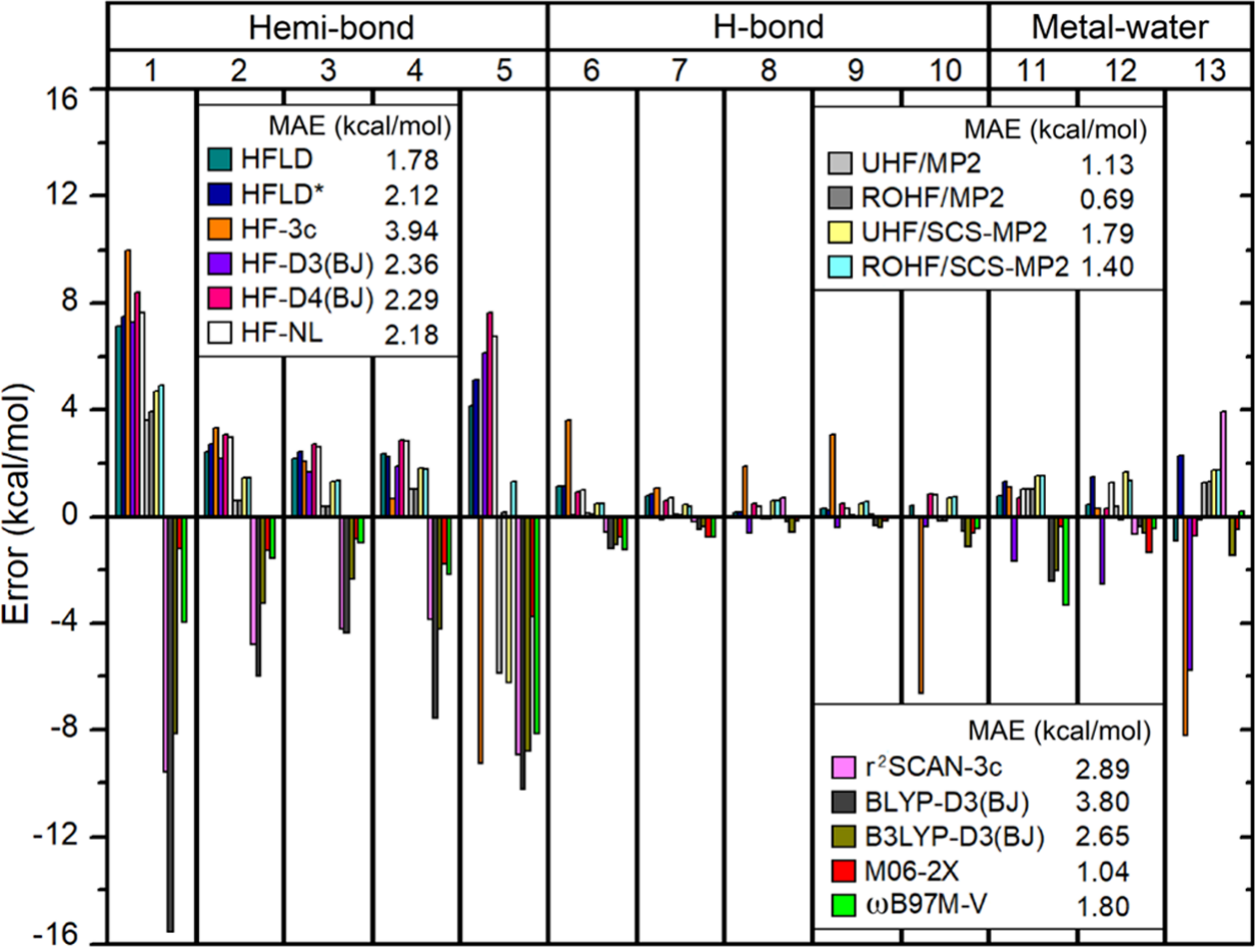
Error of interaction energy of each adduct in the TA13 set calculated with several dispersion-corrected HF and DFT methods as well as with MP2 and SCS-MP2 relative to the CCSD(T)/CBS(3/4) reference.

The interaction energy error for each adduct in the TA13 set (radical–solvent binary adducts shown in Figure 2) calculated with MP2, SCS-MP2, and several dispersion-corrected HF and DFT methods is as given in Figure 11 relative to the present CCSD(T)/CBS(3/4) reference.

All tested dispersion-corrected mean-field approaches give an MAE that is larger than 1 kcal/mol, consistent with previous extensive benchmark studies of DFT functionals.^26^ All DFT methods overestimate interaction energies, especially for hemi-bonded adducts (**1**–**5** in Figure 2). Generally speaking, pure functionals like BLYP-D3(BJ) give very large errors (MAE = 3.8 kcal/mol), while hybrid functionals like B3LYP-D3(BJ) (MAE = 2.65 kcal/mol), M06-2X (MAE = 1.04 kcal/mol), and ωB97M-V (MAE = 1.8 kcal/mol) show satisfactory accuracy. This effect can be attributed to some extent to the SIE. However, it is worth mentioning that the cost-effective r^2^SCAN-3c scheme (MAE = 2.89 kcal/mol) provides nearly the same accuracy as the popular hybrid functional B3LYP-D3(BJ).

In contrast to dispersion-corrected DFT, dispersion-corrected HF methods typically underestimate interaction energies for this set, especially for the hemi-bonded adducts interacting through an F atom (reactions 1 and 5). As discussed in Section 2.2, this effect originates from the fact that electron correlation has a deep impact on the nature of the interaction in these systems. Gold HFLD settings (MAE = ∼1.78 kcal/mol) provide an accuracy that is comparable to that of ωB97M-V, while the cost-effective HFLD* settings still show a reasonable accuracy (MAE = 2.12 kcal/mol). The other dispersion-corrected HF methods (HF-D3(BJ), HF-D4(BJ), and HF-NL) show slightly larger MAEs than HFLD*. Interestingly, the accuracy of HF-D3(BJ) is very close to that obtained with methods using more sophisticated dispersion corrections, like HF-D4(BJ) and HF-NL. In contrast, the composite HF-3c scheme provides the largest error for this set (MAE = 3.94 kcal/mol).

To summarize, gold HFLD is the best-performing dispersion-corrected HF method for the NCIs in the TA13 set, while the most accurate method for this set is ROHF/MP2 (MAE = 0.69 kcal/mol), followed by M06-2X (MAE = 1.04 kcal/mol) and UHF/MP2 (MAE = 1.13 kcal/mol). The difference between ROHF/MP2 and UHF/MP2 originates essentially from reaction 5, which is underestimated by 5.87 kcal/mol with UHF/MP2 and overestimated by 0.15 kcal/mol with ROHF/MP2.

### 4.3 Interaction Energies of the Adducts in the CARB10 Set

**Figure 12:**
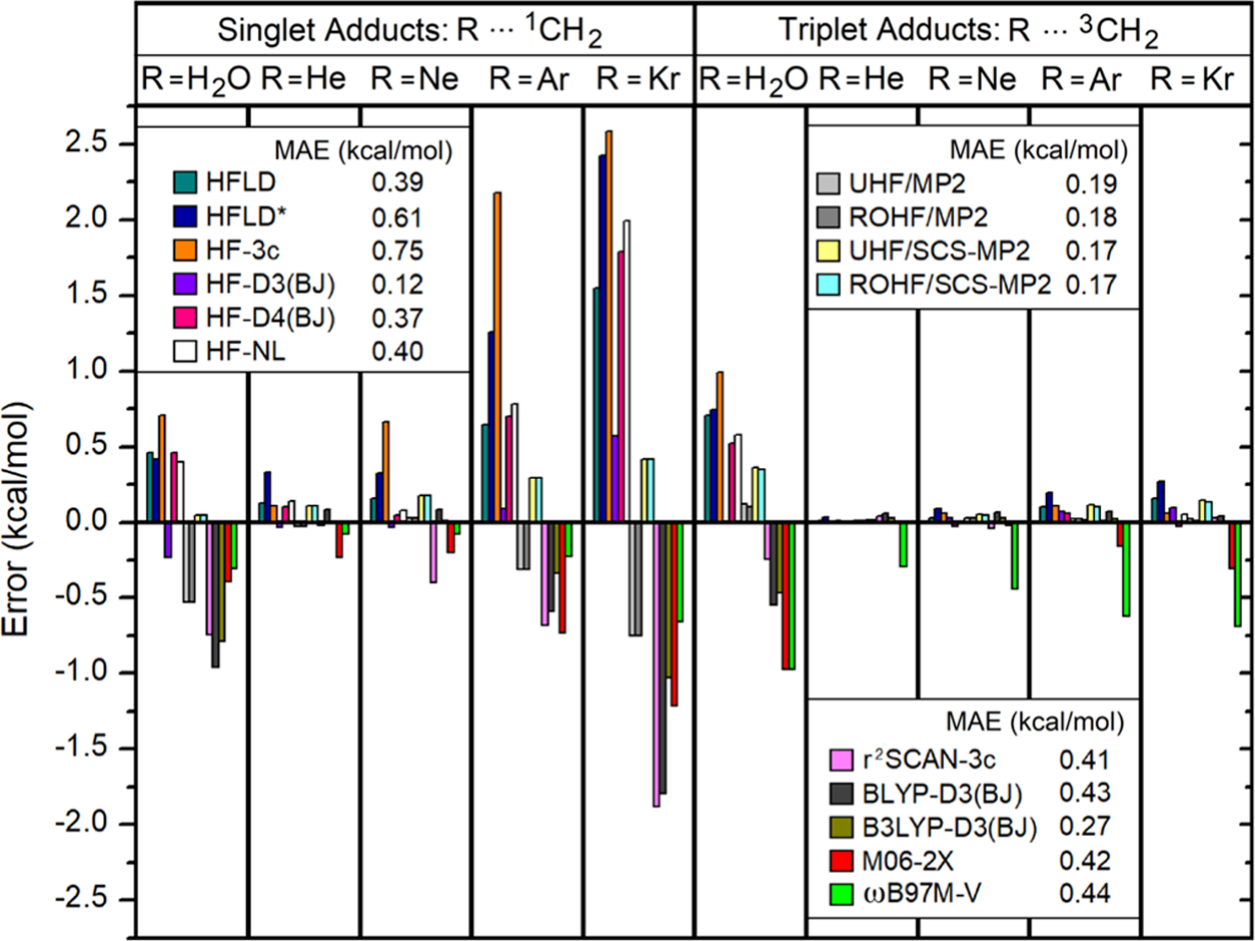
Interaction energy error for each adduct in the CARB10 set calculated with several dispersion-corrected HF and DFT methods as well as with MP2 and SCS-MP2 relative to the CCSD(T)/CBS(3/4) reference.

The interaction energy error of each adduct in the CARB10 set (Figure 3) calculated with MP2, SCS-MP2, and several dispersion-corrected HF and DFT is shown in Figure 12 relative to the present CCSD(T)/CBS(3/4) reference.

For the CARB10 set, all tested methods provide sub-kcal/mol accuracy. As in the IB8 and TA13 sets, dispersion-corrected DFT methods tend to overestimate these interaction energies due to SIE, while dispersion-corrected HF methods tend to underestimate them.^76^

In terms of MAEs, the largest (but still reasonable) error was obtained for the HF-3c scheme (0.75 kcal/mol), while the smallest error was found for HF-D3(BJ) (0.11 kcal/mol), MP2 and its variants (∼0.2 kcal/mol irrespective of the reference), and B3LYP-D3(BJ) (0.27 kcal/mol). The other tested methods, including gold HFLD, have all MAE of ∼0.4 kcal/mol. As for the IB8 and TA13 sets, the cost-effective HFLD* settings provide an ∼0.22 kcal/mol larger MAE than the gold HFLD settings.

Considering that interaction energies in the CARB10 set are smaller than those in other sets, it is also useful to discuss the mean percent error [MAE(%)] on this set. Importantly, MAE and MAE(%) correlate reasonably well for different methods, and hence the main findings just discussed hold true irrespective of the particular error metric used for the analysis. However, the analysis of the MAE(%) revealed that ωB97M-V shows errors of 20 and 460% for singlet and triplet adducts, respectively. Therefore, ωB97M-V does not describe accurately interactions involving triplet carbenes for the complexes in this set. In contrast, gold HFLD shows an MAE(%) of 44.4 and 42.4% for singlet and triplet adducts, respectively, demonstrating a satisfactory accuracy irrespective of the spin state of the system. In terms of MAE(%), the best-performing method for this set remains HF-D3, showing errors of 12.2 and 24% for singlet and triplet complexes, respectively.

### 4.4 Interaction Energies for the TTF-TCNQ Pair

**Figure 13:**
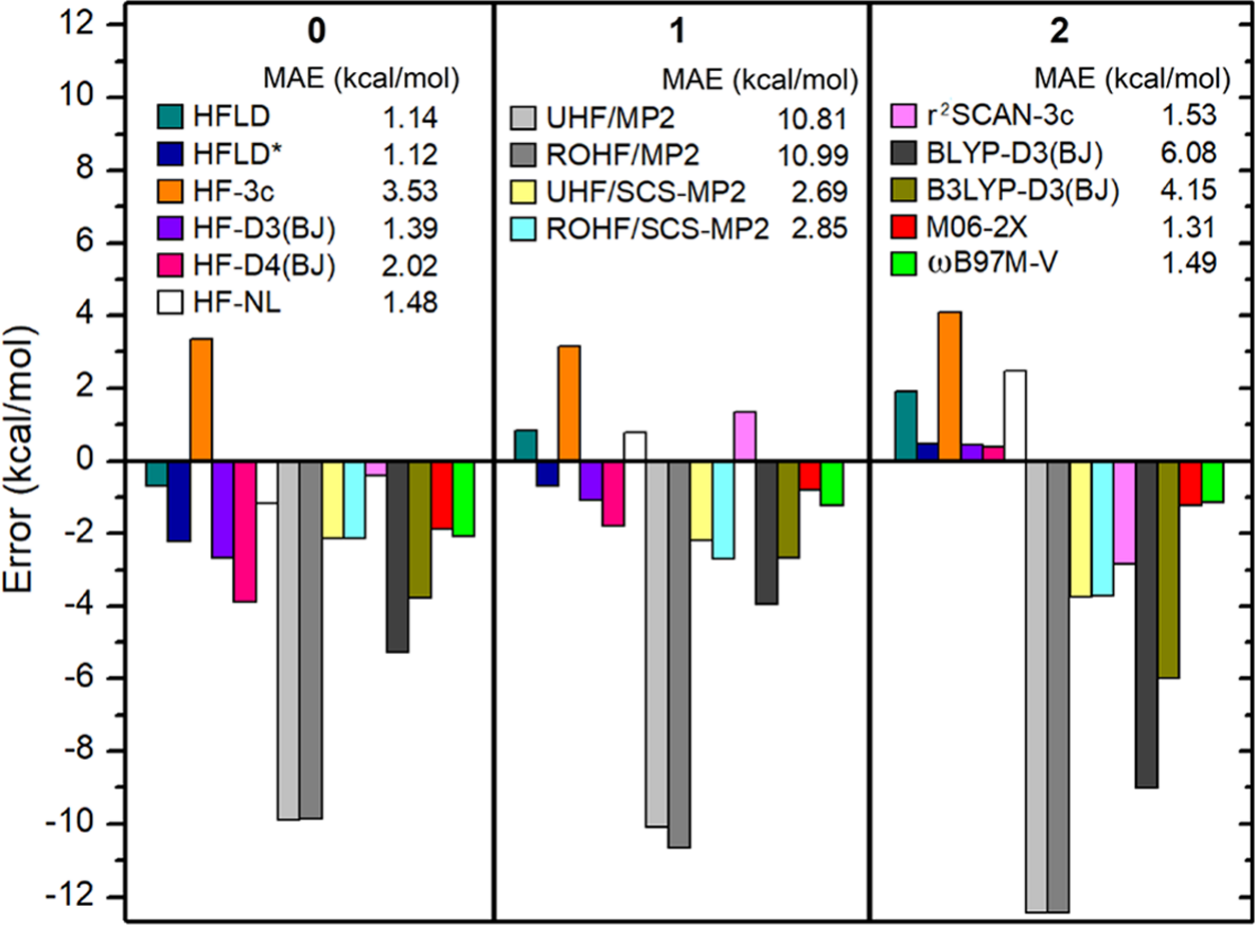
Error of interaction energy of each charge state of the TTF-TCNQ pair calculated with several dispersion-corrected HF and DFT methods as well as with MP2 and SCS-MP2 relative to the DLPNO-CCSD(T)/CPS(6/7)/CBS(3/4) reference.

The error in the stacking interaction energy of the TTF-TCNQ pair (Figure 4) for its lowest-energy singlet/neutral (**0**), one-electron oxidized doublet (**1**), and two-electron oxidized singlet (**2**) forms calculated with MP2 and SCS-MP2, and several dispersion-corrected HF and DFT methods is as given in Figure 13 relative to the present DLPNO-CCSD(T)/CPS(6/7)/CBS(3/4) reference.

For this set, HFLD and HFLD* (MAE = ∼1.1 kcal/mol) provide the most accurate interaction energies among the methods tested in this work. The satisfactory performance of the cost-effective HFLD* settings is consistent with that previously obtained on other π systems, *e.g.*, on nucleobases.^91^

For this set, the best-performing functionals are M06-2X, ωB97M-V, and r^2^SCAN-3c (MAE = 1.3–1.5 kcal/mol). The cost-effective r^2^SCAN-3c also provides a reasonable accuracy. Interestingly BLYP-D3/D4 and B3LYP-D3/D4 provide MAEs between 3 and 6 kcal/mol. However, BLYP-NL and B3LYP-NL provide errors that are similar to those obtained with the other best-performing functionals, with MAEs of 1.89 and 1.09 kcal/mol, respectively (see TTF-TCNQ-DFT-HFD sheet in the Supporting Information).

Finally, it is worth emphasizing here that, for such dispersion-bound adducts, MP2 fails dramatically, irrespective of the reference determinant used (MAE = ∼11 kcal/mol). The MAE associated with SCS-MP2 is much smaller but still noticeable (MAE = ∼2.8 kcal/mol). Interestingly, the very expensive OO variants of MP2 and SCS-MP2 both have typically twice larger errors than the corresponding non-OO variants (see TTF-TCNQ-MP2 sheet in the Supporting Information). Therefore, MP2 and its variants are not appropriate for such challenging stacking interactions.

### 4.5 Overview of the Accuracy of the Methods

**Figure 14:**
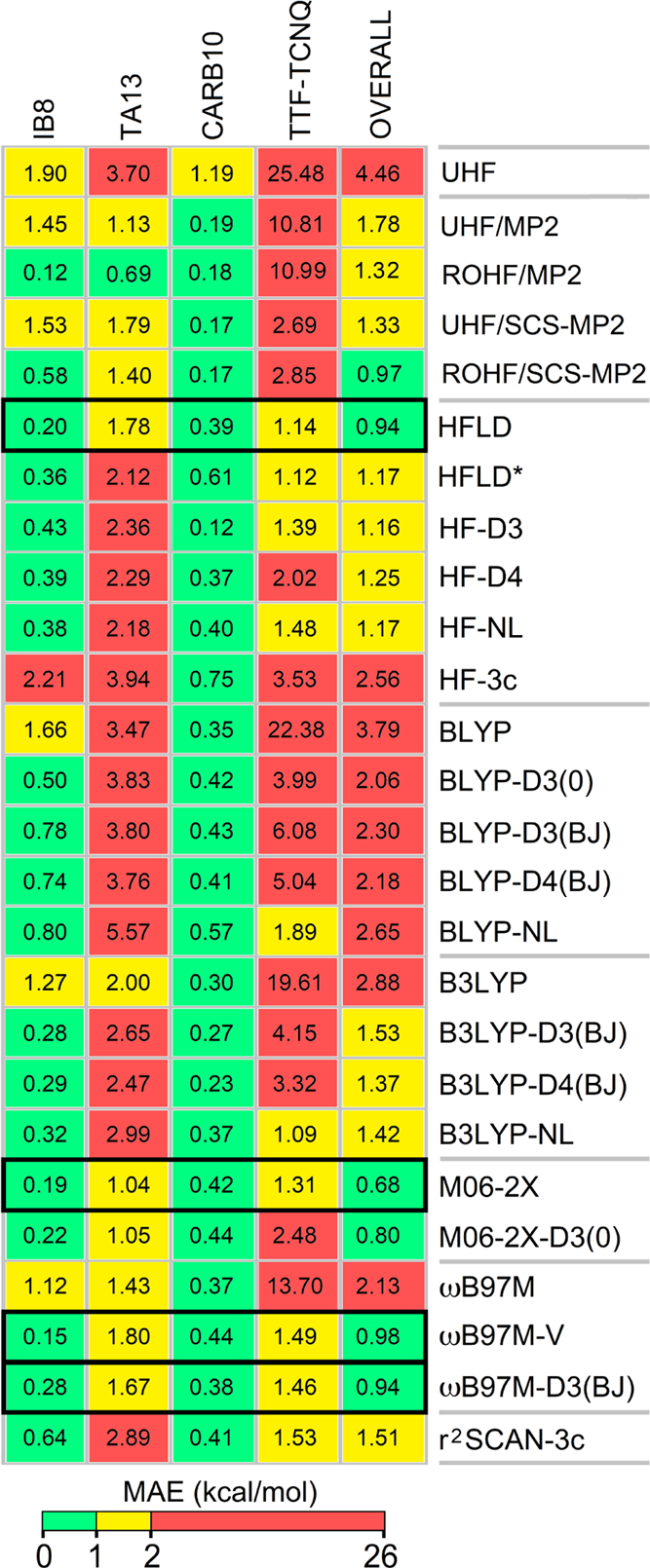
MAEs of the methods for interaction energies of IB8, TA13, CARB10, and TTF-TCNQ benchmark sets as well those over all benchmark sets (color scheme for MAEs: green, less than 1 kcal/mol; yellow, between 1 and 2 kcal/mol; and red, above 2 kcal/mol).

Figure 14 shows the MAEs obtained for HF, MP2, and its variants; several dispersion-corrected HF methods; and several dispersion-uncorrected and -corrected DFT functionals for all of the benchmark sets studied in this work. MAEs were colored according to their range: green, less than 1 kcal/mol; yellow, between 1 and 2 kcal/mol; and red, above 2 kcal/mol.

The best-performing methods over all benchmark sets are gold HFLD, M06-2X, ωB9M-V, and ωB9M-D3(BJ). These methods show MAEs that are less than 2 kcal/mol for all sets, and an overall MAE that is less than 1 kcal/mol. All other tested methods have at least one benchmark set for which they fail (MAE > 2 kcal/mol). Note that HFLD* settings still provide a reasonable accuracy for all benchmark sets.

### 4.6 Efficiency of HFLD and Complementary Analysis Tools: The Case of Di(1-Adamantyl)Carbene in Water

**Figure 15:**
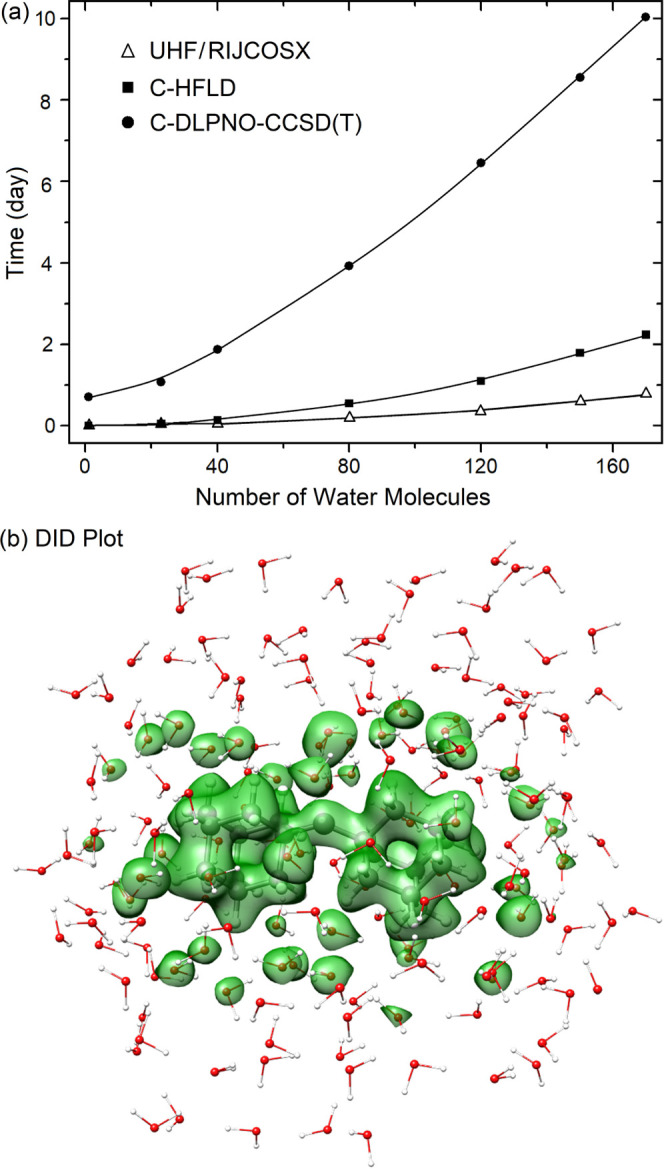
(a) Total wall time associated with the reference (UHF/RIJCOSX) and correlation parts of HFLD/NormalPNO* (C-HFLD) and DLPNO-CCSD(T_0_)/NormalPNO* (C-DLPNO-CCSD(T)) BSSE-corrected interaction energy calculations for the triplet di(1-adamantyl)carbene interacting with varying number of water molecules. The def2-TZVP(-f) basis set was used in all cases. In all cases, two cores from a single cluster node equipped with four Intel Xeon CPUs were used. (b) Corresponding dispersion interaction density (DID) plot at the HFLD* level. The density isosurface value was set to 0.01 kcal/(mol bohr^3^).

Figure 15a shows the wall time associated with the reference and correlation parts of HFLD* and DLPNO-CCSD(T_0_) interaction energy calculations for the triplet DAC interacting with an increasing number of water molecules. While HFLD and DLPNO-CCSD(T_0_) are both near linear scaling methods, they show different prefactors. Hence, wall times are significantly smaller for HFLD than for DLPNO-CCSD(T_0_). When the system size increases, the difference in the computational time of these two methods increases significantly.

For several closed-shell molecular systems, it was previously shown that the computational cost of the correlation part of HFLD (C-HFLD) is roughly the same as that of the SCF in standard mean-field approaches.^88^ A similar trend is obtained here for DAC interacting with up to ∼40 water molecules (system size: 171 atoms) as shown in Figure 15a. However, for larger systems, the C-HFLD becomes slightly more expensive than HF or hybrid DFT. For example, for the largest solvated DAC model (system size: 561 atoms), an interaction energy calculation takes 0.77 day with UHF/RIJCOSX/def2-TZVP(-f) and 1.52 day with ωB97M-V/RIJCOSX/def2-TZVP(-f), while a C-HFLD calculation at the HFLD* level takes 2.24 days using the same computational resources (see Figure 15a).

It is important to note here that the LD term in the HFLD scheme corresponds to the physical LD energy contribution, and it can thus be used to study LD effects in large and complex systems in great detail. In addition, the “dispersion interaction density” (DID) function^77,135^ can be used to provide a detailed spatial analysis of the LD energy obtained with the HFLD scheme. For example, the DID plot associated with the DAC–water interaction at the HFLD* level (see Figure 15b) demonstrates that LD is only significant between DAC and its first solvation shell.

In addition, if combined with the LED scheme, the HFLD approach can be used to quantify the other physical contibutions to the interaction (*e.g*., electrostatic, exchange, and electronic preparation), as recently shown for the interaction of the base pairs in a DNA duplex model.^91^ Thus, the open-shell variant of the HFLD scheme opens up unprecedented opportunities for the accurate quantification and analysis of NCIs in large and complex systems.

## 5 Conclusions

The open-shell variant of the nonempirical HFLD method for the study NCIs in open-shell molecular systems was presented. This approach allows us to quantify NCIs in large and complex systems, while providing at the same time an in-depth physical insight into their nature. On challenging benchmark sets for NCIs involving open-shell molecules, the HFLD scheme was found to be the most accurate dispersion-corrected HF method among those tested in this work. Its accuracy is superior to that obtained for the popular dispersion-corrected B3LYP-D3/D4/NL schemes, and it is comparable to that found for the best-performing exchange-correlation functionals, *i.e.*, M06-2X, ωB97M-V, and ωB97M-D3(BJ).

## Supporting Information Available

Input file structures, Cartesian coordinates of all adducts, details of CBS extrapolation, as well as detailed energetics and error analyses (XLSX)
